# Supplementary material for: Impact of a multilevel HIV prevention programme on adolescent girls and young women’s empowerment for risk reduction: A mixed methods evaluation in Kenya
Source: PLOS Glob Public Health. 2025 Jun 23;5(6):e0004151. doi: 10.1371/journal.pgph.0004151 (PMC12185012; doi:10.1371/journal.pgph.0004151)
Supplement: Attachment — Submitted filename: Rebuttal letter_19.12.24.docx [file pgph.0004151.s005.docx]

**Response to editors' and reviewers’ comments**

RE: Manuscript number PGPH-D-24-02060

“Impact of the DREAMS Partnership on Adolescent Girls and Young Women’s Empowerment for HIV Risk Reduction: A Mixed Methods Evaluation in Kenya”.

Dear Editor,

Thank you for the opportunity to revise and resubmit our manuscript, based on the helpful reviews. We are pleased to submit the revised manuscript, along with a point by point response to all comments from the editors and reviewers. We appreciate the time accorded to review this manuscript and the comments you sent have helped to enrich this paper.

**Point-by-point responses to the reviewers’ comments**

**Section: Editors comments**

1. Please include a complete copy of PLOS’ questionnaire on inclusivity in global research in your revised manuscript. Our policy for research in this area aims to improve transparency in the reporting of research performed outside of researchers’ own country or community. The policy applies to researchers who have travelled to a different country to conduct research, research with Indigenous populations or their lands, and research on cultural artefacts. The questionnaire can also be requested at the journal’s discretion for any other submissions, even if these conditions are not met.  Please find more information on the policy and a link to download a blank copy of the questionnaire here: <https://journals.plos.org/globalpublichealth/s/best-practices-in-research-reporting>. Please upload a completed version of your questionnaire as Supporting Information when you resubmit your manuscript.

**Response:** This form has been completed and included with our resubmission

2. Your current Financial Disclosure states, “The funders had no role in study design, data collection and analysis, decision to publish, or preparation of the manuscript.”. However, your funding information on the submission form indicates that you received funding from “Bill and Melinda Gates Foundation INV-027727 Abdhalah Ziraba”. Please indicate by return email the full and correct funding information for your study and confirm the order in which funding contributions should appear. Please be sure to indicate whether the funders played any role in the study design, data collection and analysis, decision to publish, or preparation of the manuscript.

**Response**: The funding was provided by the Bill and Melinda Gates Foundation REF INV-027727, in a grant led by Abdhalah Ziraba (Principal Investigator) at APHRC. Dr Ziraba is a recipient of the funding and not a representative of the funder. The funders at the Foundation had no role in the design, evaluation, analysis or interpretation of findings**.**

3. In the online submission form, you indicated that "Data sources will be available upon request from the authors.".

All PLOS journals now require all data underlying the findings described in their manuscript to be freely available to other researchers, either

1. In a public repository,

2. Within the manuscript itself, or

3. Uploaded as supplementary information.

This policy applies to all data except where public deposition would breach compliance with the protocol approved by your research ethics board. If your data cannot be made publicly available for ethical or legal reasons (e.g., public availability would compromise patient privacy), please explain your reasons by return email and your exemption request will be escalated to the editor for approval. Your exemption request will be handled independently and will not hold up the peer review process, but will need to be resolved should your manuscript be accepted for publication. One of the Editorial team will then be in touch if there are any issues.

**Response**: Final anonymized quantitative datasets are made available at the African Population and Health Research Center (APHRC’s) Microdata Portal (<https://microdataportal.aphrc.org/index.php/catalog>).

4. Figure 1: Please confirm whether you drew the images / clip-art within the figure panels by hand. If you did not draw the images, please provide (a) a link to the source of the images or icons and their license / terms of use; or (b) written permission from the copyright holder to publish the images or icons under our CC-BY 4.0 license. Alternatively, you may replace the images with open source alternatives. See these open source resources you may use to replace images / clip-art:

- [https://commons.wikimedia.org](https://commons.wikimedia.org/)

- <https://openclipart.org/>

**Response:** Figure 1 has now been removed as it is adequately described in text. We have also provided a reference for the conceptual framework, to credit the earlier thinking of Kabeer (1999).

5. We have noticed that you have a list of Supporting Information legends in your manuscript. However, the uploaded files are figures which already uploaded in the file inventory. Kindly remove the citation of "Supporting Information".

**Response.** This has been removed

6. While the study intervention to address HIV among younger population is commendable, the entire paper reads as a feedback to the DREAMS project. Authors need to describe the scalability and sustainability of this intervention and other similar interventions to empower young population infected with HIV in resource poor and conflict settings

**Response:** In the Introduction and Discussion we have now included points to broaden the relevance of this paper’s purpose and findings, e.g., to apply the findings beyond Kenya to DREAMS investments in 14 other countries; for the increasing number of investments in multi-component or combination HIV prevention; and for other efforts to incorporate empowerment strategies within HIV prevention.

Please note that DREAMS was not implemented in conflict settings, so we have not addressed replication or scalability in such contexts.

7. Themes and sub-themes are insufficiently described

**Response:** We have added a table to provide a clearer description of themes and sub-themes in the results section.

8. Enthusiastic consent and sexual coercion is simplified in the study results description. Please describe in detail and explain the challenges in teaching such concepts to young population in the discussion

**Response**. We have now edited the Results to be more explicit that the right to refuse sex was expressed by many participants, especially in the theme of bodily integrity. We explain how tangible and intangible resources enabled some young women to assert this right. However, we also noted the failure of some institutional structures to support this right, e.g., participants cited a lack of action by police in cases of rape. Reflecting on this section (enabling institutional structures) in the Discussion, we have now added that we did have evidence of whether DREAMS taught consent concepts to males in the same communities.

9. In Nairobi did any any child refuse to participate in the study? Of those who participated without parental consent, what were challenges? Can these children be termed as emancipated due to abuse or neglect or absent parents? What additional protection measures were taken when assent was obtained from these children to ensure autonomy was not compromised?

**Response**: In Nairobi, there were 4 refusals to consent among adolescents <18 years, who were actively followed up in the 2022 (the fourth) round of data collection. Their decision was respected and these individuals were withdrawn from the study. For those who agreed to participate in an additional round to the evaluation, parents were informed and verbally agreed but written consent from a parent/guardian was waived by the ethics committee on the basis of sensitivity of topics asked related to sexual behavior. During the informed consent process, additional protection measures included assurances of their right to stop or withdraw at any time (none did so). We did not experience challenges in the recruitment as all had participated in earlier rounds of the evaluation (2017-2019) and were familiar with the study procedures and data protection measures.

10. Clarify the need for Figure 1 when the BMGF Document is already cited in the article

Response: This figure has been removed

11. Child abuse incidents are being described in this study. What are the preventive measures taken by investigators to address vicarious trauma among local investigators?

**Response.** The research team comprised trained research assistants with experience in interviewing adolescents, including questions related to violence (emotional, physical and sexual). They also had a team leader and research officer/supervisor who routinely organized meetings to share feedback on experiences in the field. Research assistants received appropriate guidance, routine field breaks to rest, and counseling where requested. We have now added this point in the Methods.

12. Please provide the CRediT statement for the authors

**Response**: This statement has been added to the manuscript

13. Statistical analysis needs further description in methods and clarify whether it is a mixed methods study design

**Response:** More details on statistical analysis has now been provided. We have also clarified that this is a mixed methods study design, in both the Abstract and Methods.

Reviewer 1 comments

14. Reviewer #1: 1. Baseline data in Table 1 is not clear, what is the unit? Is it frequency, or percentage?
    I believe it is n, but it will be great if it is mentioned.
    Also, the total number of samples should be mentioned at the table.

**Response**. We have edited Table 1 to clarify that the unit is the number of participants in qualitative research activities

15. The quantitative aspect of the study can be best explained in a tabular form.

**Response**: We appreciate this suggestion and have now added supplementary tables IA and 1B to provide further detail for the results in Figures 2 and 3. We have retained Figures 2 and 3 in the main manuscript for a visual summary of the differences between groups.

**Reviewer 2 comments**

Reviewer #2: Abstract

The abstract states the study's elements—introduction, methods, results, and conclusions—facilitating a coherent progression for readers.

You can consider explicitly writing the research questions or objectives, such as “This study aims to evaluate the extent to which DREAMS interventions have promoted the empowerment of adolescent girls and young women (AGYW) in Kenya”. Consider including it in the abstract as well as in the introduction.

**Response**. We have now included this statement in the Abstract, as suggested

The statement "we discovered insufficient evidence of a causal effect" (line 36) can be further clarified. What is the meaning of "weak evidence" in this context? Does it pertain to statistically negligible outcomes, minimal effect sizes, or other constraints?

**Response:** This language has been corrected to state that “we found **statistical evidence** of a causal impact on social support and self-efficacy among adolescent girls” (In the Results chapter we describe the strength of this evidence in terms of probability and confidence intervals.)

Authors may want to mention any prominent themes or patterns that arose from the interviews and conversations, beyond specific examples such as "pooling resources together" (line 42).

**Response**: We have mentioned themes related to collective action, solidarity, and group saving skills for financial autonomy. We have removed specific examples due to limitations in the word count in the abstract.

The abstract references empowerment precursors such as "resources, agency, and institutional structures" (line 29), which are essential notions in empowerment theory. It may be beneficial to reference the theoretical framework underpinning this study, whether it is the specific DREAMS framework or another recognised empowerment paradigm.

**Responses**: In this section of the Abstract, we have now stated that we were guided by Kabeer’s framework of women’s empowerment

16. Introduction

It would be beneficial to offer a concise explanation or operational definition of "gender-transformative". How does the study conceptualise "gender-transformative" in relation to DREAMS? (line 83)
The word "structural determinants" (line 69) might benefit from a concise explanation or an illustrative example for people unfamiliar with the idea.

**Response**: In the introduction, we have explained further that ‘gender-transformative’ approaches are explicit in redressing gender inequalities and removing structural barriers to empowerment.

17. Methods

The methods section is detailed and robust, but with clearer descriptions of the quantitative analysis, more integration of the conceptual framework, it would better demonstrate the rigor of the research. The section on how participants were selected—both for the overall cohort and for the qualitative components—demonstrates methodological rigor.

**Response**: We have now elaborated on the approach to quantitative analysis, and how we were guided by the conceptual framework to integrate data across sources

The operationalisation of social support and self-efficacy is clear, but it would be helpful to have a short description of the specific questions that were used to measure them.

**Response:** A short description of specific questions has been added including a reference to the previous analysis in which we describe the methodology in detail (in our analysis of earlier DREAMS evaluation data).

The statistical methods used for causal analysis could be explained in more depth. How were the different possible outcomes (early invitees vs. later invitees) analysed?

**Response**: A more detailed description of the causal analyses has been added.

The conceptual model of women and girls’ empowerment, based on Kabeer’s framework, is mentioned, but not fully included in the description of the methods. It would benefit readers to show how the methods were chosen, interview guides were made, and the coding process was carried out in qualitative analysis.

**Response:** We have added information on what informed selection of the methods and how the coding process was carried out by the Kabeer’s framework constructs.

The part on results gives a complete and well-organized picture of how DREAMS has affected the empowerment of AGYW. The section can be made even stronger by showing how outcomes vary by the age group (Adolescents & young women), making the statistical analysis clearer and describing more about the institutional hurdles.

**Response**: Although age group disaggregated analyses were done for quantitative analyses, this was not applied to the qualitative aspects. We have existing information on the age group outcomes for both the social support and self-efficacy outcomes.

18. Discussion

The discussion section presents a thorough interpretation of the findings. The conversation will provide even more insightful information for upcoming programmes if it places more emphasis on quantifiable outcomes, examines institutional hurdles in greater detail, and makes age specific recommendations.

**Response:** We appreciate this feedback. We have now added a section in the Discussion with more emphasis on quantifiable outcomes, institutional barriers, and age-specific recommendations. We feel that these additions have helped to broaden the scope and relevance of the Discussion, per Point 6 of the Editors’ comments above.
