# Supplementary material for: Impact of a multilevel HIV prevention programme on adolescent girls and young women’s empowerment for risk reduction: A mixed methods evaluation in Kenya
Source: PLOS Glob Public Health. 2025 Jun 23;5(6):e0004151. doi: 10.1371/journal.pgph.0004151 (PMC12185012; doi:10.1371/journal.pgph.0004151)
Supplement: Attachment — Submitted filename: Rebuttal letter_21.02.25.docx [file pgph.0004151.s006.docx]

**Response to editors' and reviewers’ comments**

RE: Manuscript number PGPH-D-24-02060R1

“Impact of the DREAMS Partnership on Adolescent Girls and Young Women’s Empowerment for HIV Risk Reduction: A Mixed Methods Evaluation in Kenya”.

Dear Editor,

Thank you for the opportunity to revise and resubmit our manuscript, based on the helpful reviews. We are pleased to submit the revised manuscript, along with a point by point response to all comments from the editors and reviewers. We appreciate the time accorded to review this manuscript and the comments you sent have helped to enrich this paper.

**Point-by-point responses to the reviewers’ comments**

**Section: Editors comments**

1. Introduction: A prominent example of multi-level programming to prevent HIV risk among adolescent
   85 girls and young women is the DREAMS Partnership (Determined, Resilient, Empowered, AIDS-free,
   86 Mentored, and Safe) led by PEPFAR and private sector partners.
   These details especially the funder details can be put in reference and not in the manuscript.

Response: Thanks for the comment, we have deleted the section “led by PEPFAR and private sector partners”. Although the DREAMS program is PEPFAR supported, this evaluation was funded by a different funder(Gates Foundation) and this information is already in the funder details.

2. A conceptual framework would be much helpful for readers to understand the themes visually

Response: Thanks for this comment, we have already elaborated Kabeer’s women empowerment conceptual framework that guided the analysis of the study (lines 205-214). The summary table on themes arising from the analysis also presents the same. We feel this is adequate for the reader.
