# Supplementary material for: Impact of a multilevel HIV prevention programme on adolescent girls and young women’s empowerment for risk reduction: A mixed methods evaluation in Kenya
Source: PLOS Glob Public Health. 2025 Jun 23;5(6):e0004151. doi: 10.1371/journal.pgph.0004151 (PMC12185012; doi:10.1371/journal.pgph.0004151)
Supplement: Attachment — Submitted filename: Rebuttal letter_22.04.25.docx [file pgph.0004151.s007.docx]

**Response to editors' and reviewers’ comments**

RE: Manuscript number PGPH-D-24-02060R2

“Impact of the DREAMS Partnership on Adolescent Girls and Young Women’s Empowerment for HIV Risk Reduction: A Mixed Methods Evaluation in Kenya”.

Dear Editor,

Thank you for the opportunity to revise and resubmit our manuscript, based on the helpful reviews. We are pleased to submit the revised manuscript, along with a point by point response to all comments from the editors and reviewers. We appreciate the time accorded to review this manuscript and the comments you sent have helped to enrich this paper.

**Point-by-point responses to the reviewers’ comments**

**Section: Editors comments**

Thank you for your prompt and adequate response to the queries. The paper looks brilliant.

I kindly request you to elaborate further in the "Evaluation research contexts: DREAMS in Kenya" section by shifting the DREAMS description in the introduction section to methods section

In the introduction please stick to the following sub sections

- HIV and social determinants of health

- Psychological aspects of HIV

- Rights, dignity and legal aspects and any other aspects relevant to the study

**Response**

Thank you for the comment. We have moved most of the section on DREAMS to the evaluation of research contexts section; we have only left a short paragraph explaining the objective of the paper. The shortened introduction covers the suggested sections on HIV and social determinants of health, and psychological aspects of HIV. The rights and legal aspects were not a major focus in this paper, but we have added a short description in the methods section.

There is a need to add a study settings section in methods in which you elaborate the prevalence of HIV in relevant populations in the study setting, any ongoing geopolitical issues, conflicts in the study area, legal issues, health care access available in the study area (with regards to availability of ART, PREP, PEP etc, literacy, child marriages rates and other relevant information concerning the study area for the readers to connect the dots.

**Response**: We have added information on HIV prevalence in both study contexts with a focus on adolescents and young people and information on barriers to access to prevention services such as PrEP in the study setting section

Since the intervention has definitely significant impact in PLHIV, authors can add a subsection at the end of the discussion on relevance for policy making in resource-constrained settings for HIV prevention and holistic care.

**Response:** We feel the DREAMS interventions focus on the prevention of HIV incidence, and this paper does not focus on impact among PLHIV. We have not considered this suggestion.
