# Supplementary material for: Impact of a multilevel HIV prevention programme on adolescent girls and young women’s empowerment for risk reduction: A mixed methods evaluation in Kenya
Source: PLOS Glob Public Health. 2025 Jun 23;5(6):e0004151. doi: 10.1371/journal.pgph.0004151 (PMC12185012; doi:10.1371/journal.pgph.0004151)
Supplement: Attachment — Submitted filename: Rebuttal letter_14.05.25.docx [file pgph.0004151.s008.docx]

**Response to editors' and reviewers’ comments**

RE: Manuscript number PGPH-D-24-02060R3

”.

Dear Editor,

Thank you for yet another opportunity to revise and resubmit our manuscript, based on the helpful reviews. We are pleased to submit the revised manuscript, along with a point by point response to all comments from the editors and reviewers. We appreciate the time accorded to review this manuscript.

**Point-by-point responses to the reviewers’ comments**

**Section: Editors comments**

Since the authors feel that the paper does not focus on impact among PLHIV, the relevance of paper in PGPH journal is doubtful. Authors are requested to elaborate on how the paper is in alignment with the journal which aims to address global inequities and creating impactful research in public health.

The title of the paper could be changed to " Impact of a multilevel programming and empowerment intervention for HIV risk reduction among adolescent girls and young women: A mixed methods evaluation in Kenya". DREAMS can be explained in the paper

**Response.** Thanks for the suggested new title, we have made a slight adaptation and stated it as

“Impact of a multilevel HIV prevention programme on adolescent girls and young women’s empowerment for risk reduction: a mixed methods evaluation in Kenya.”

“We feel this paper’s focus on the large gender and age inequities in HIV risk is consistent with the aims of *PLOS Global Public Health* to address global health inequities. In all countries with generalised HIV epidemics, adolescent girls and young women experience the highest rates of infection (greater than their male peers and older groups). The paper investigates the impact of gender empowerment strategies embedded within HIV prevention, with the ultimate aim to empower HIV-negative young women and reduce the number living with HIV. Thus, while DREAMS does not focus directly on PLHIV (for example, holistic care and HIV treatment are not part of the DREAMS interventions), we have explained the relevance to HIV prevention and gender equity within the Introduction and Discussion.”
